# Resting TcPO2 levels decrease during liner wear in persons with a transtibial amputation

**DOI:** 10.1371/journal.pone.0239930

**Published:** 2020-09-28

**Authors:** Martin C. Berli, Michèle Jundt-Ecker, Margrit R. Meier, Michael Hofer, Madlaina Schöni, Tobias Götschi, Ilker Uçkay, Thomas Böni, Felix W. A. Waibel

**Affiliations:** 1 Department of Orthopaedic Surgery, Balgrist University Hospital, Zurich, Switzerland; 2 ConReha GmbH, Buttikon, Switzerland; 3 Rehaklinik Bellikon, Bellikon, Switzerland; 4 Department of Orthopaedic Surgery, Institute for Biomechanics, University of Zurich, ETH Zurich, Balgrist Campus, Zurich, Switzerland; National University of Ireland Galway, Galway, Ireland, IRELAND

## Abstract

**Background:**

In our clinic, a substantial number of patients present with transtibial residual limb pain of no specific somatic origin. Silicone liner induced tissue compression may reduce blood flow, possibly causing residual limb pain. Thus, as a first step we investigated if the liner itself has an effect on transcutaneous oxygen pressure (TcPO2).

**Methods:**

Persons with unilateral transtibial amputation and residual limb pain of unknown origin were included. Medical history, including residual limb pain, was recorded, and the SF-36 administered. Resting TcPO2 levels were measured in the supine position and without a liner at 0, 10, 20 and 30 minutes using two sensors: one placed in the Transverse plane over the tip of the Tibia End (= TTE), the other placed in the Sagittal plane, distally over the Peroneal Compartment (= SPC). Measurements were repeated with specially prepared liners avoiding additional pressure due to sensor placement. Statistical analyses were performed using SPSS.

**Results:**

Twenty persons (9 women, 11 men) with a mean age of 68.65 years (range 47–86 years) participated. The transtibial amputation occurred on average 43 months prior to study entry (range 3–119 months). With liner wear, both sensors measured TcPO2 levels that were significantly lower than those measured without a liner (TTE: p < 0.001; SPC: p = 0.002) after 10, 20 and 30 minutes. No significant differences were found between TcPO2 levels over time between the sensors. There were no significant associations between TcPO2 levels and pain, smoking status, age, duration of daily liner use, mobility level, and revision history.

**Conclusion:**

Resting TcPO2 levels decreased significantly while wearing a liner alone, without a prosthetic socket. Further studies are required to investigate the effect of liner wear on exercise TcPO2 levels.

## Introduction

A certified prosthetist and orthotist’s or an orthopaedic surgeon’s daily practice regularly encounters a person with a transtibial amputation (TTA) who presents with pain in the residual limb while wearing their prosthesis [1]. Accordingly, a thorough investigation of possible reasons for the pain needs to be conducted jointly by the responsible CPO and surgeon to facilitate prosthesis use. However, in our daily practice in a tertiary referral center for orthopedic surgery with a specialized unit for diabetic feet, amputations, and prosthetic and orthotic aftercare, we are confronted with a substantial number of patients in whom the thorough collaborative investigation did not reveal a specific somatic pathology as the cause of pain.

As part of the collaborative investigation outlined above, we identify whether the patient suffers from residual limb pain (RLP) or phantom limb pain [2, 3]. Whereas phantom limb pain is experienced as coming from the amputated limb, RLP is defined as pain present in the distal part of the residuum. RLP represents acute nociceptive pain [4, 5]. Most reported pain still present six months after an amputation is RLP, with many cases related to a poorly fitting prosthesis [6, 7]. Both RLP and phantom limb pain negatively affect a patient’s overall satisfaction with life [4].

Dwornik et al. described as many as 23 possible causes of RLP (Table 1) [6]. Most causes represent mechanical problems: either intrinsic anatomical aberrations in the residual limb, or extrinsic aberrations caused by prosthetic misfittings, liners that have been used too long, poor liner handling, or silicone allergies. All of these mechanical aberrations can be resolved by treating the underlying mechanical challenges [6].

**Table 1:** Reasons for residual limb pain and corresponding therapeutic options, adapted from Dwornik et al. [6].

THERAPEUTIC OPTIONS	
Mainly surgical therapy	Mainly conservative therapy
Exostoses	Insufficient prosthetic fitting
PAD	Adjacent joint degeneration
Osteitis / Osteomyelitis / Abscess	Psychological influence
Poor bone shaping of residual limb	Skin allergies
Poor bone length balancing (e.g. fibula overlength)	Residual limb width undulation
	Thrombosis
	Hyperhidrosis
Both therapeutic options	
Scar tissue / Mesh grafts	
Skin lesions / ulcers / socket border nodules	
Soft tissue surplus	
Haematoma, Seroma	
Neuroma / Nerves not being removed	

Another reported cause of RLP is peripheral arterial disease (PAD) [6]. Unfortunately, the literature on PAD-related perfusion adaption in the residual limb and its effect on RLP is scarce. Two case reports [8, 9] connect a reduction in residual limb perfusion with RLP and highlight the need for an increased focus on this issue. The natural causes of PAD can slowly lead to residual limb claudication and RLP. However, external compression of the residual limb by the prosthetic socket system may also cause a reduction in residual limb blood flow, particularly at the local microperfusion level, thereby potentially resulting in RLP.

The prosthetic socket is the interface between the human and the mechanical parts of the prosthetic system. A snug fit of the prosthetic socket is required, as body weight is also transferred through soft tissue compression. Most prosthetic socket systems use a liner [10–12], for total surface bearing with a more equal pressure distribution across the surface of the residual limb [13] based on the hydrostatic volume principle [11, 12]. The manufacturer of the liner used in this study states silicone liners generate a linear residual limb volume reduction of 3% to 5% distally to 0% proximally. The socket volume, however, is reduced in the opposite direction, from 3% to 5% proximally to 0% distally. Thus, a constant hydrostatic pressure of 3% to 5% is generated on the residual limb. Depending on the condition of the tissue, this hydrostatic pressure corresponds to a working pressure of 40–70 mmHg, thus creating an elevated pressure on the residual limb at rest, even though weight bearing and the resulting increased pressures due to external loading have not yet occurred. Thus the “starting” pressure elevation induced by a liner is a potential explanation for RLP, as it may diminish local perfusion and induce vascular compression.

The determination of transcutaneous oxygen pressure (TcPO2) values is a well-established method for assessing local tissue perfusion. It involves determination of the tension of oxygen diffusing from local skin with an electrode that serves as a gas sensor. With skin warming to 43 to 44°C and capillary dilatation in the skin for induction of arterial blood inflow, the oxygen tension in this skin region approaches the peripheral tissue arterial blood oxygen tension. This permits noninvasive evaluation of microcirculation in a local region through analysis of TcPO2 [14–16]. TcPO2 is commonly used as a predictor for wound healing potential in ulcer cases or for the determination of adequate amputation levels [17–25]. For example, a TcPO2 value of <40 mmHg has been associated with poor healing outcomes in persons with a transtibial amputation and a two-fold higher risk in requiring conversion to a transfemoral amputation [20].

The literature on perfusion adaption in persons with a transtibial amputation is scarce. Only a few studies have analyzed TcPO2 levels in residual limbs, but possible relationships between TcPO2 levels and pain have not been investigated.

Residual limb TcPO2 in persons with a lower limb amputation is considerably reduced. Rink et al compared resting TcPO2 levels in five persons with a transtibial amptuation (four due to trauma, one due to infection) while wearing and not wearing a liner [26]. It is unknown whether the participants had any undelying conditions such as PAD or were suffering from RLP. TcPO2 was measured on the posterior calf side. Resting TcPO2 with liner in place (41.0 ± 10.4 mmHg) was reduced by approximately 30% when compared to TcPO2 levels without a liner (57.8 ± 9.2 mmHg). In able-bodied controls this reduction was even greater, representing a near 50% drop.

Bramley et al compared TcPO2 levels between the amputated and non-amputated side in a person with a transtibial amputation [27]. Pressure was applied to the non-amputated side through an inflatable cuff, with pressure magnitudes similar to the pressure experienced with a pneumatic post-amputation mobility aid, to simulate the pressure experienced by the residual limb through a transtibial prosthesis. TcPO2 was measured at three sites: at the patella tendon, the lateral calf, and the posterior calf. The pressure was gradually increased through the inflatable cuff to reach 60 mmHg after 50 minutes. TcPO2 levels demonstrated more than 25% change from baseline at all three sites, with lateral calf and patella tendon showing more than 75% change from baseline. The visual estimated rate of change in TcPO2 levels on the posterior and lateral calf sites appeared to be greater on the residual limb than on the contralateral, non-ampuated side. Once pressure was released, an overshoot of up to 25% change from baseline was recorded. Ten minutes after the cuff had been removed, none of the sites’ readings had returned to starting levels. Unfortunately, no actual TcPO2 readings were presented, and it is not known if the participant experienced RLP.

Given the information presented, our primary study goal was to determine alterations in resting TcPO2 values during liner usage in the population we see most: persons with PAD, and those who experience persistent RLP with no somatic pathology as its cause. The secondary study goals were to evaluate possible correlations of resting TcPO2 values with smoking status, age, sex, mobility level, duration of daily liner usage, and surgical revision history after amputation.

## Methods

### Clinical setting

Balgrist University Hospital is a tertiary referral center for orthopedic surgery with a specialized unit for the management of diabetic feet, orthopedic complications of PAD, amputations, and prosthetic and orthotic aftercare. The unit consists of three fellowship-trained orthopedic surgeons, two internists, one infectious disease specialist, and a crew of more than 10 certified prosthetist and orthotists. Affiliations exist with the departments of angiology and vascular surgery at the University Hospital Zürich. The interdisciplinary collaborative examination of a patient’s situation is conducted with a combination of team members customized to the requirements of the individual patient. At least one orthopedic surgeon and one certified prosthetist and orthotist are always involved.

### Study participants

The study was approved by the local ethics committee "Kantonale Ethikkommission Zürich, Stampfenbachstrasse 121, 8090 Zürich Switzerland" (BASEC-No. 2016–00395). All study participants provided informed written consent. Patients who underwent a TTA at our institution between 2007 and 2017 were eligible for study participation. All TTAs were performed by or under the direct supervision of a senior orthopedic surgeon. Amputations were performed as described by Burgess and Zettl [28].

Recruitment of potential study participants took place between March and September 2017. Inclusion criteria were: age older than 18 years, having had a TTA, being fitted with a definitive prosthesis that includes a prosthetic liner system, daily use of the prosthesis for at least two hours per day, and RLP for longer than three hours per day. Exclusion criteria were: somatic causes for RLP (according to Table 1), radiculopathy, bony tumor as an amputation indication, and drug or alcohol abuse.

All participants completed a standardized medical history, which included the amputation level, reason for amputation, presence/absence of PAD and/or diabetes mellitus, history of smoking, level of mobility (K-Level) as assessed by the Amputee Mobility Predictor [29], intensity of pain when using the prosthesis (evaluated using the Prosthesis Evaluation Questionnaire, Group 2, question I, which assesses pain with a standard visual analogue score [VAS]) [30], and the hours of daily prosthesis use. After recording the standardized medical history, the participant completed the SF-36 questionnaire.

### TcPO2 test setup

The utilized TcPO2 measurement device (TCM4 Series ETX391-880R0263N015 2014, Radiometer, Bronshoj, Denmark, last maintained on July 27^th^, 2015) was validated by Groullier et al. in 2006 [31]. Clark-type sensors were incorporated in the device for measurement of TcPO2 [32]. For standardization purposes, the certified prosthetist and orthotist gave only the *“Iceross comfort cushion liner”* (Össur, Reykjavik, Iceland) to all study participants during the time of measurement. Each participant received the correct liner size for his or her residual limb. Liner thickness at the points of measurement was 7 mm.

**Fig 1:**
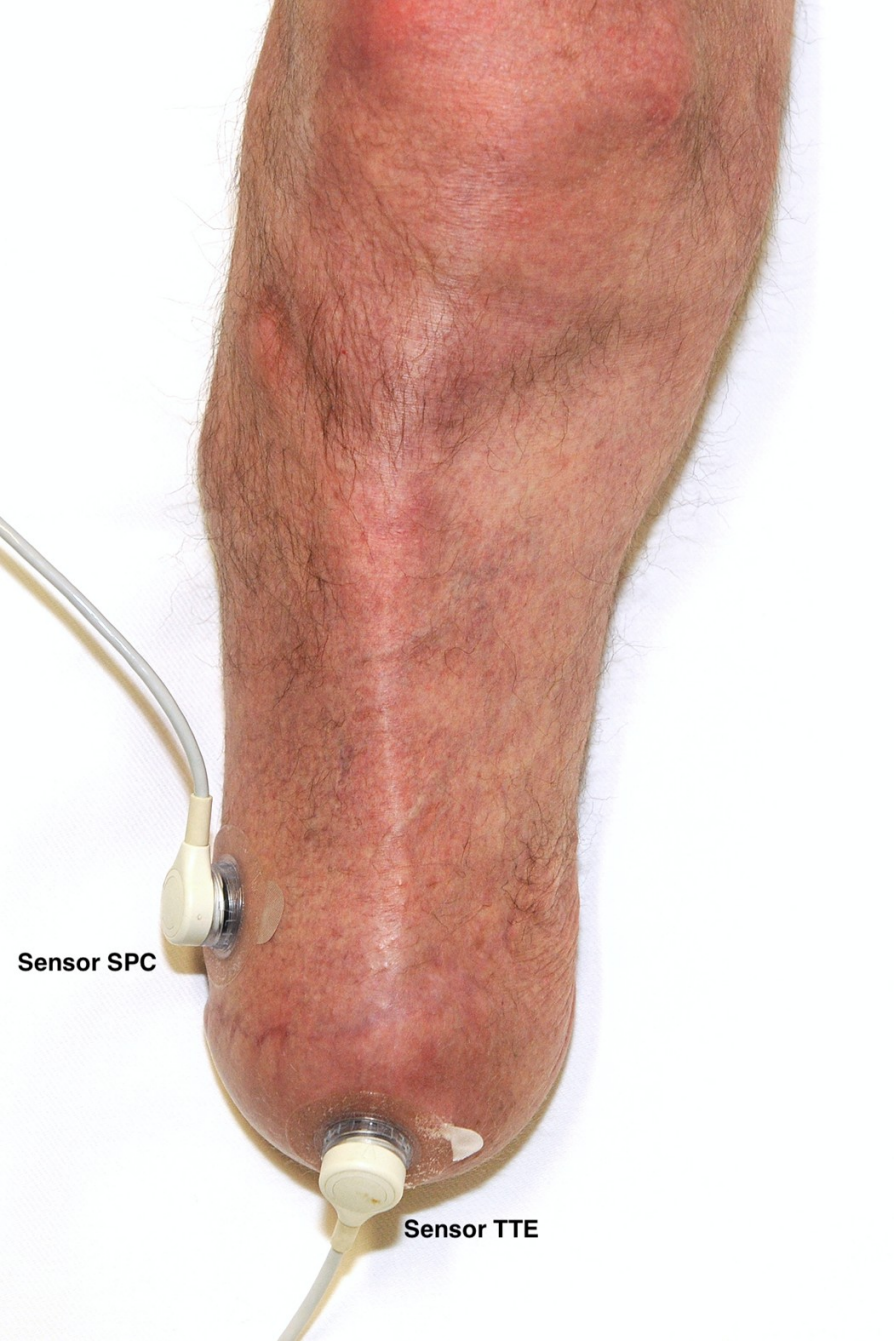
Test setup, anterior view. Sensor TTE placed over the top of the tibia at the most distal part of the posterior Burgess flap, Sensor SPC placed over the peroneal compartment at the most distal part of the anterior Burgess flap.

**Fig 2:**
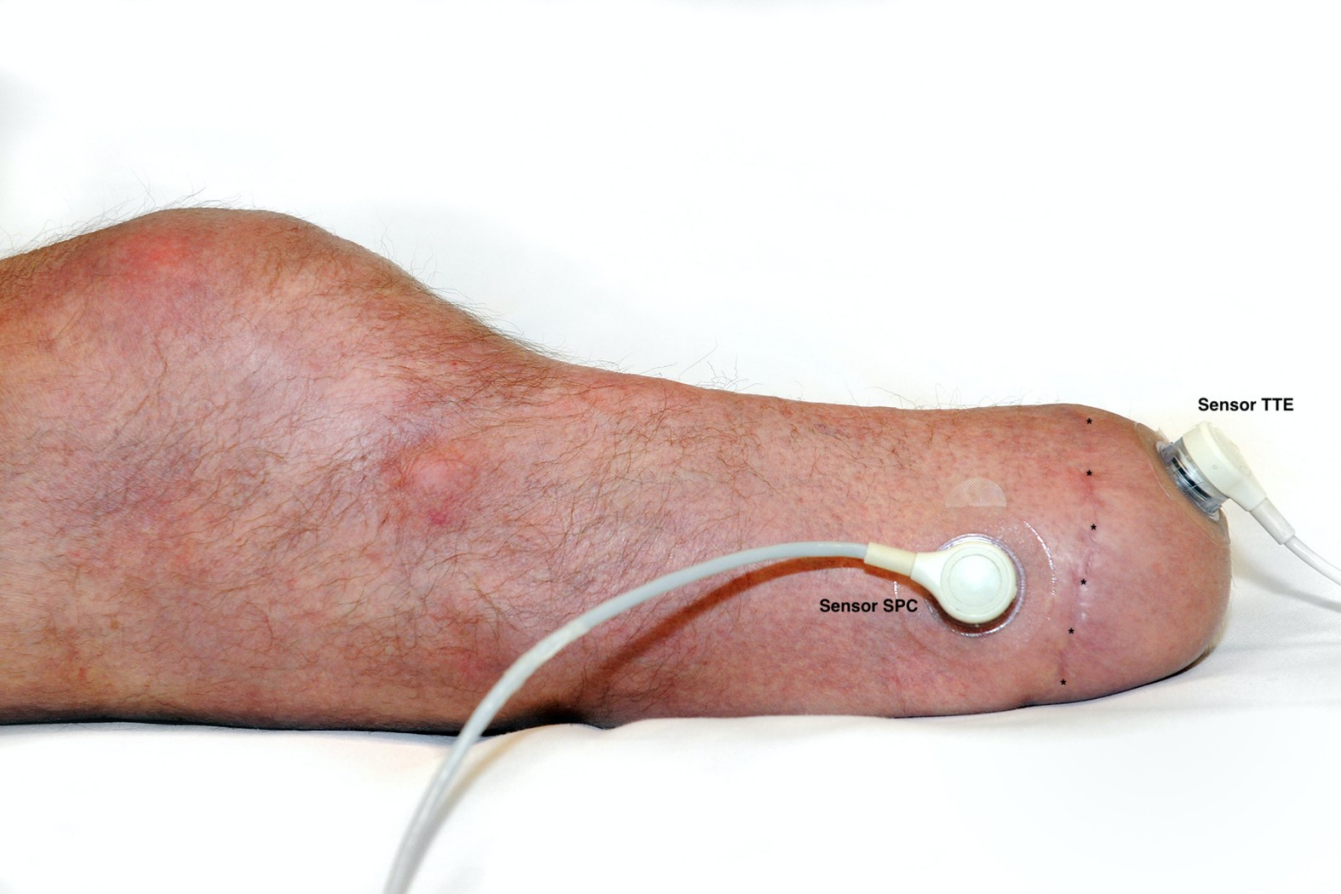
Test setup, lateral view. Same sensor placements as described in Fig 1. The scar (*) depicts the dividing line between the anterior and posterior Burgess flaps and therefore illustrates the choice of sensor placement at the most distal parts of both flaps.

For the purpose of TcPO2 measurements, study participants were placed in a supine position described as the best discrimination position for limb ischemia [33, 34]. The knee was fully extended during the entire measurement process. All measurements were made during rest, i.e. no activities were undertaken before the test session. The sensors were calibrated and heated automatically by the TCM4 measurement device. Two sensor electrodes were used and placed directly on the skin of the residual limb following skin cleaning with alcohol and drying with a gauze pad. Contact liquid (Radiometer, Bronshoj, Denmark) was applied in the space between the skin and the sensor in accordance with the instruction manual. One sensor was placed in the Transverse plane over the tip of the Tibia End (sensor TTE) where the remaining gastrocnemius flap is fixed, and the second sensor was placed in the Sagittal plane distally over the Peroneal Compartment (sensor SPC) (Figs 1 and 2). These locations were chosen as they represent the most distal parts of the posterior skin flap (sensor TTE) and the most distal part of the anterior skin flap (sensor SPC). In these locations, perfusion is most restricted, and they represent the worst-case perfusion scenario.

Measurements were taken in the same order for all participants, i.e. the same protocol was followed for each of the study participants. The test session began with the person being tested laying supine on a clinical bench, both legs extended and the residual limb exposed (no prosthesis or liner). After an acclimation period, in which in accordance with the instruction manual, calibration, heating of the probes to 44°C and the skin by the sensors for 15 minutes were performed, the first baseline measurement (T0) was recorded without the liner. Thereafter, three additional measurements without the liner were taken at 10, 20, and 30 minutes for a total of four measurements (T0, T10, T20, T30). The same procedure was repeated, but this time while wearing the liner. To prevent pressure-related measurement errors as well as pressure ulcers, small holes corresponding to the size of the sensors were punched out of the liner such that no external pressure was applied to the sensors. Punching the holes into the liner took roughly 1 minute, during which the participant continued to lay supine on the clinical bench. None of the sensors were removed. TcPO2 data were retrieved visually.

### Statistical analyses

Statistical analyses were carried out using SPSS (Version 23/IBM Corp. Chicago Illinois). A power analysis (alpha error set at 0.05) revealed a minimum sample size of 13 participants. The corresponding underlying assumption was a TcPO2 level of 35 mmHg for Condition 1 (not wearing a liner) and a TcPO2 level of 50 mmHg for Condition 2 (wearing a liner), with a standard deviation of 15 mmHg. Because visual data inspection showed a departure from normal distribution of the TcPO2 values for both sensors and conditions, a Shapiro-Wilk normality test was applied and confirmed this initial impression. We therefore employed nonparametric statistical testing. Wilcoxon signed-rank tests were used to examine the TcPO2 levels between the two conditions that were measured at the different time points. To test for a possible time effect on the two tested conditions, a nonparametric repeated-measures model (Friedman’s test) was used to investigate absolute differences in TcPO2 levels within each condition. Potential differential effects of the two conditions on the different TcPO2 levels for dichotomous variables were evaluated using Mann-Whitney U tests. Spearman rank correlation analyses assessed associations between numerical demographic parameters and TcPO2 levels at baseline.

## Results

### Participants

Twenty participants (11 men, 9 women) with a mean age of 68.65 years (range 47–86 years) took part in the study. Participants’ demographics are shown in Table 2. Mean time from amputation to TcPO2 measurements was 43 (range 3–119) months.

**Table 2:** Participants’ demographics.

Demographics	Number of participants (n = 20)
Men : Women	11 : 9
Site involved (right : left)	15 : 5
Mean age (years)	68.65 (range 47–86) years
Mean time from TTA	43 (range 3–119) months
PAD	16 (80%)
Active Smoking	7 (35%)
Mean daily use of prosthesis	11.45 (range 2–16) hours
K-Level*	
1	3 (15%)
2	8 (40%)
3	6 (30%)
4	3 (15%)
Reason for amputation:	
Infection after elective surgery	9 (45%)
Foot osteomyelitis	7 (35%)
Charcot arthropathy	4 (20%)

* based on Amputee Mobility Predictor (AMP).

In nine participants the reason for amputation was wound complications following previous surgical foot procedures: four arthrodeses and five minor foot amputations. Additional reasons for amputation were osteomyelitis due to pressure ulcers in seven participants, and complications of Charcot arthropathy (infected pressure ulcers with subsequent osteomyelitis) in four participants. Four participants also required revision surgery after amputation. No ulcers were present when TcPO2 measurements were performed.

### SF-36

**Fig 3:**
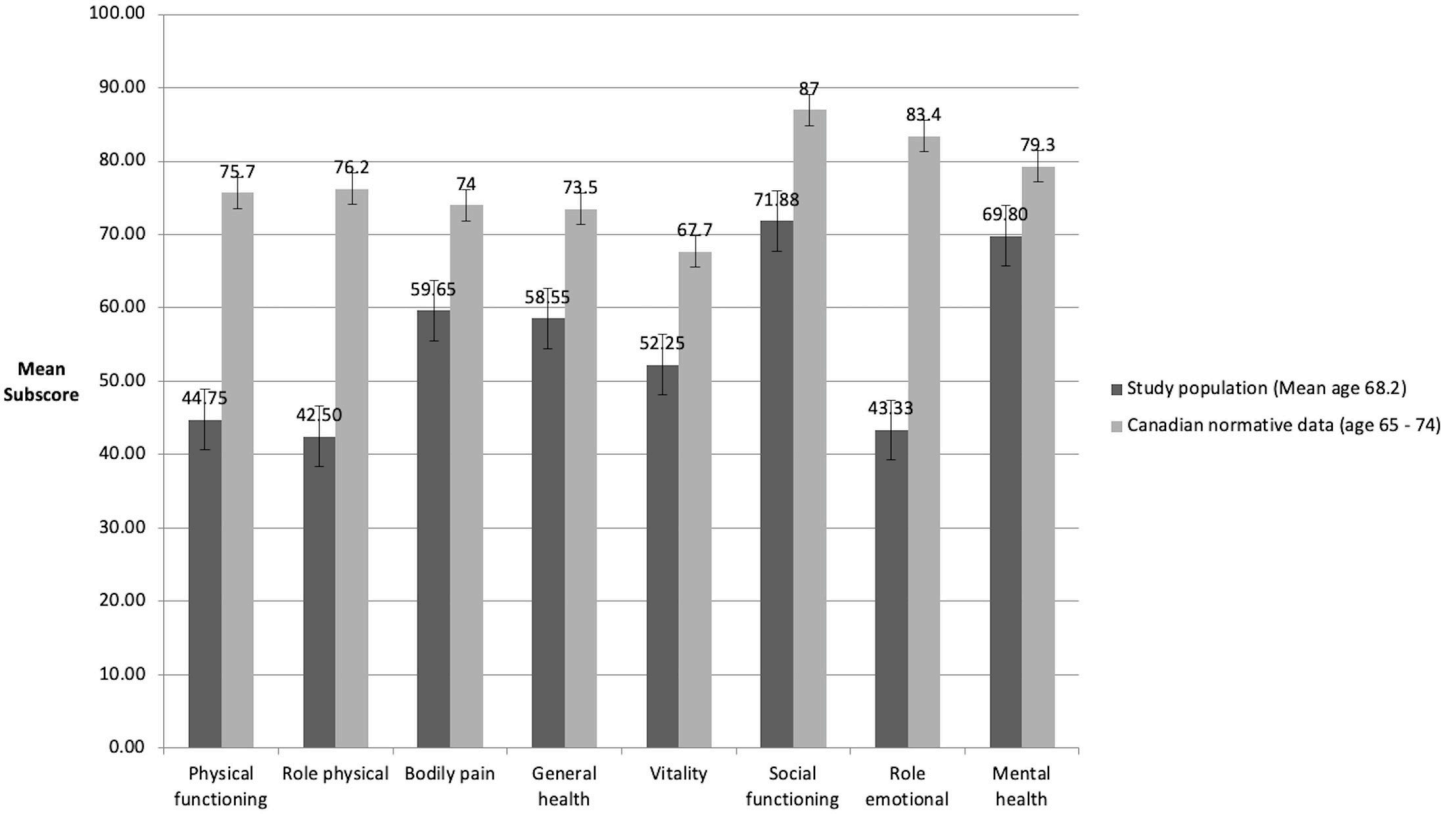
SF-36 scores of study participants in comparison with Canadian normative data [35].

**Fig 4:**
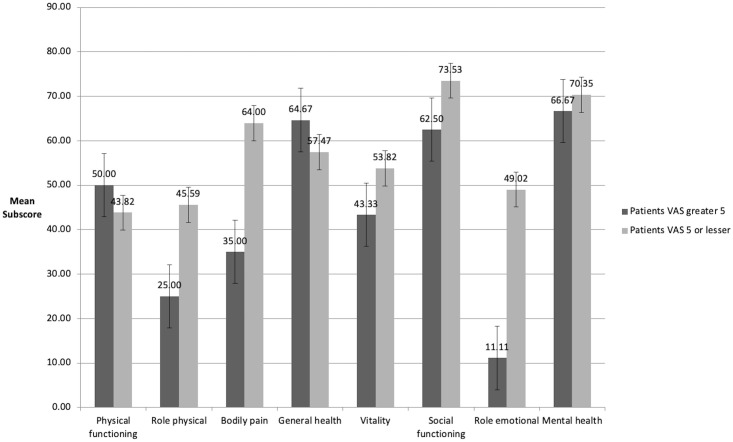
Comparison of SF-36 scores of participants with residual limb pain with a VAS score ≥5 and participants with a VAS score <5.

All SF-36 results are shown in Figs 3 and 4. Compared to age-related normative data [35], study participants scored lower in all sub-scores (Fig 3). In addition, participants who experienced pain at VAS ≥5 scored higher in physical functioning and general health, but lower in all other SF-36 sub-scores (Fig 4). Comparing the SF-36 results with the K-Levels revealed that participants with a K3-Level or higher rated their physical and mental health lower than participants with K2-Level or less.

### Resting TcPO2 and time

**Fig 5:**
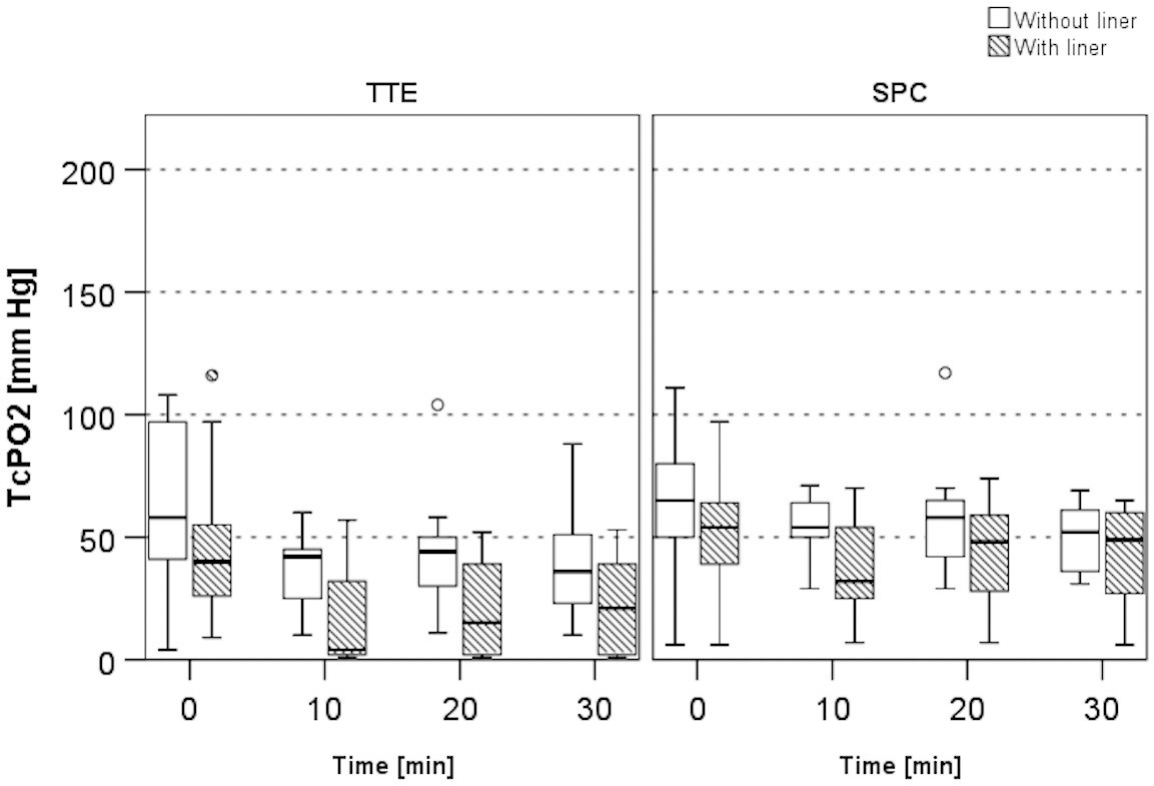
TcPO2 levels for Sensors TTE and SPC. Median TcPO2 levels with and without liner wear for time points T0, T10, T20, and T30 minutes. Median, inter-quartile range and total range (and outliers if applicable) are shown.

At the initial TcPO2 measurement T0, no statistically significant differences were detected for both sensors between conditions 1 and 2, although TcPO2 levels were lower at both sensor sites with condition 2. All resting TcPO2 values decreased significantly after 10, 20 and 30 minutes of wearing a liner (Table 3, Fig 5).

**Table 3:** TcPO2 in mmHg for both sensors at all time points, with and without liner wear.

Sensor	Time	TcPO2 (mmHg) Median (IQR)		*p*-value^a^
		Without liner	With liner	
TTE	0	58 (56)	40 (29)	0.067
TTE	10	42 (20)	4 (28)	<0.001
TTE	20	44 (20)	15 (37)	<0.001
TTE	30	36 2(8)	21 (37)	<0.001
SPC	0	65 (30)	54 (25)	0.25
SPC	10	54 (14)	32 (29)	<0.001
SPC	20	58 (23)	48 (31)	0.001
SPC	30	52 (25)	49 (33)	0.003

^a^ Wilcoxon Signed Ranks Test

The Friedman test for time on the paired differences in resting TcPO2 was non-significant for both sensors: TTE (χ2(3) = 2.578, p = 0.461) and SPC (χ2(3) = 2.132, p = 0.545). These findings imply that the median resting TcPO2 level between the two conditions is not differently affected by time.

### Resting TcPO2 and variables pain, age, daily prosthetic use, and time from amputation

**Fig 6:**
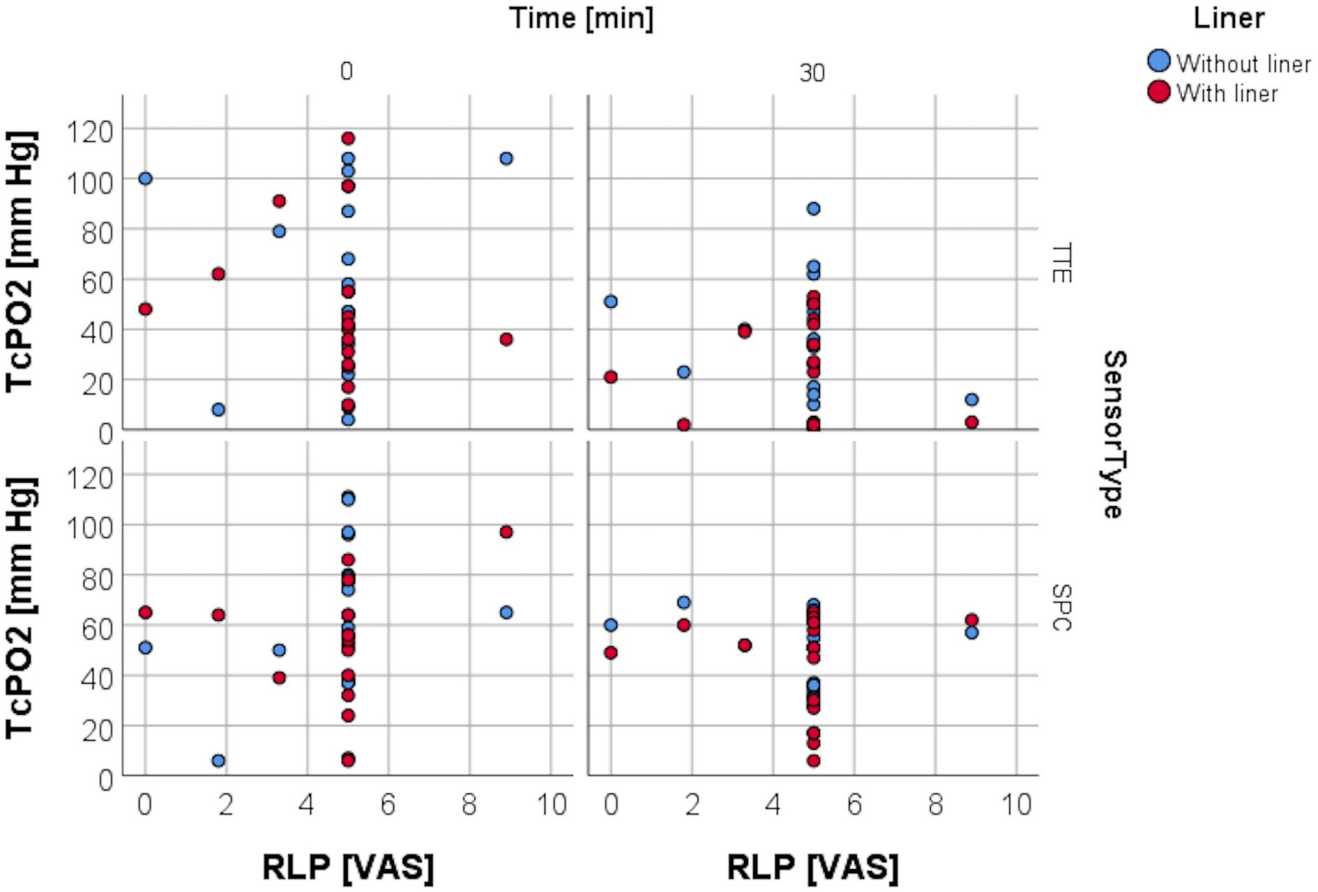
TcPO2 levels and RLP. Scattergram depicting distribution of TcPO2 levels with and without liner wear depending on the amount of residual limb pain.

**Fig 7:**
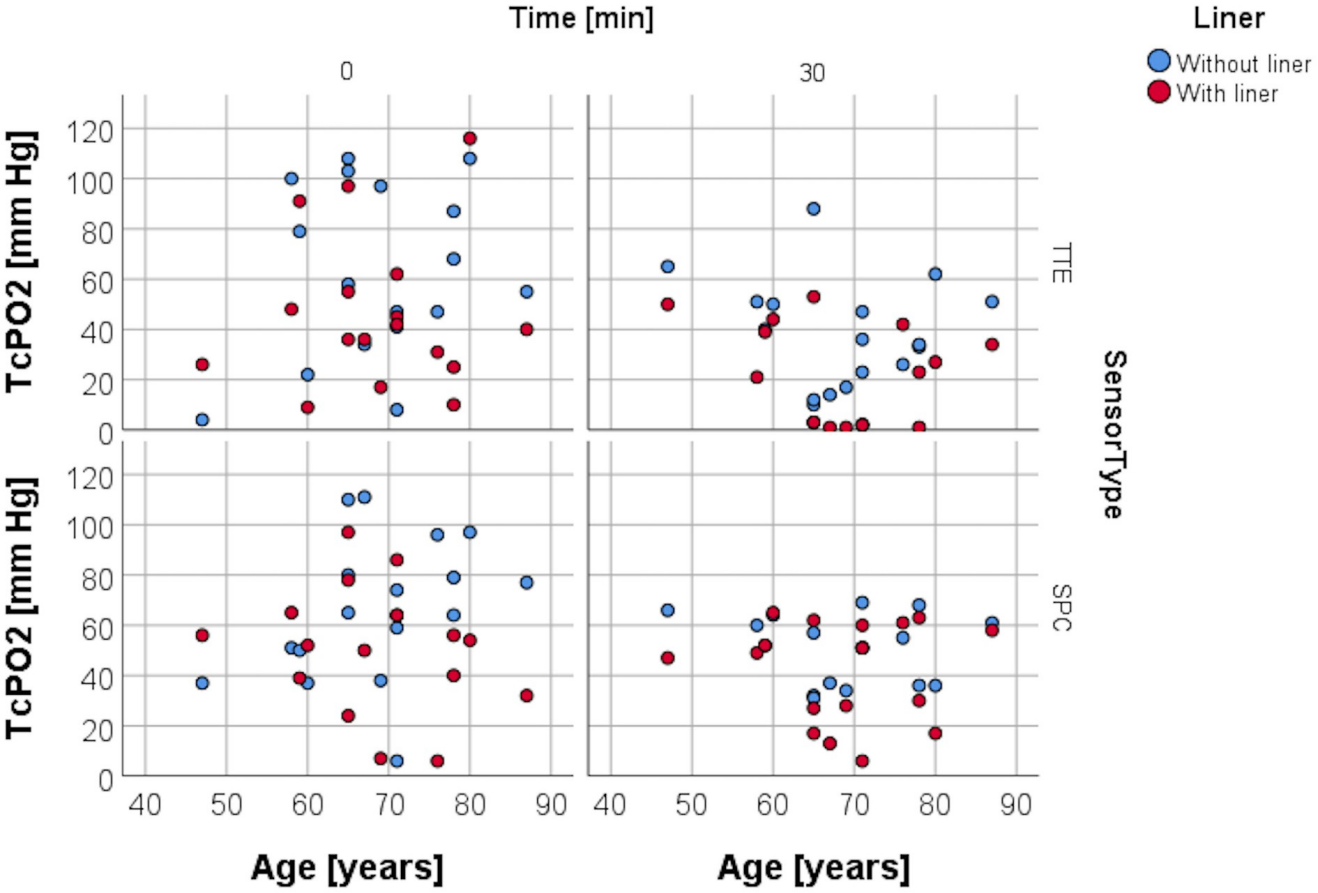
TcPO2 levels and age. Scattergram depicting distribution of TcPO2 levels with and without liner wear depending on the age of the participants.

**Fig 8:**
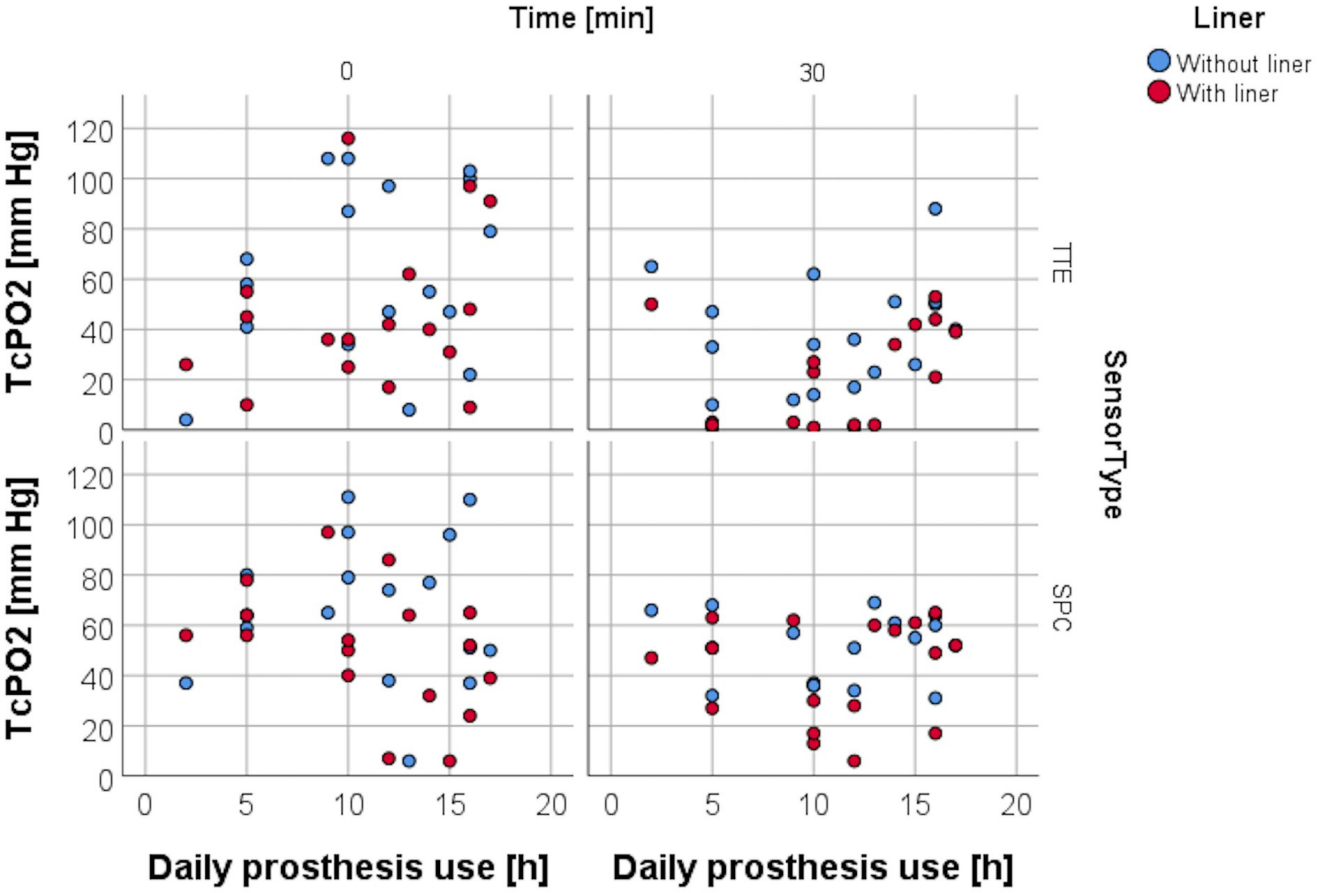
TcPO2 levels and daily prosthetic use. Scattergram depicting distribution of TcPO2 levels with and without liner wear depending on daily prosthetic use.

**Fig 9:**
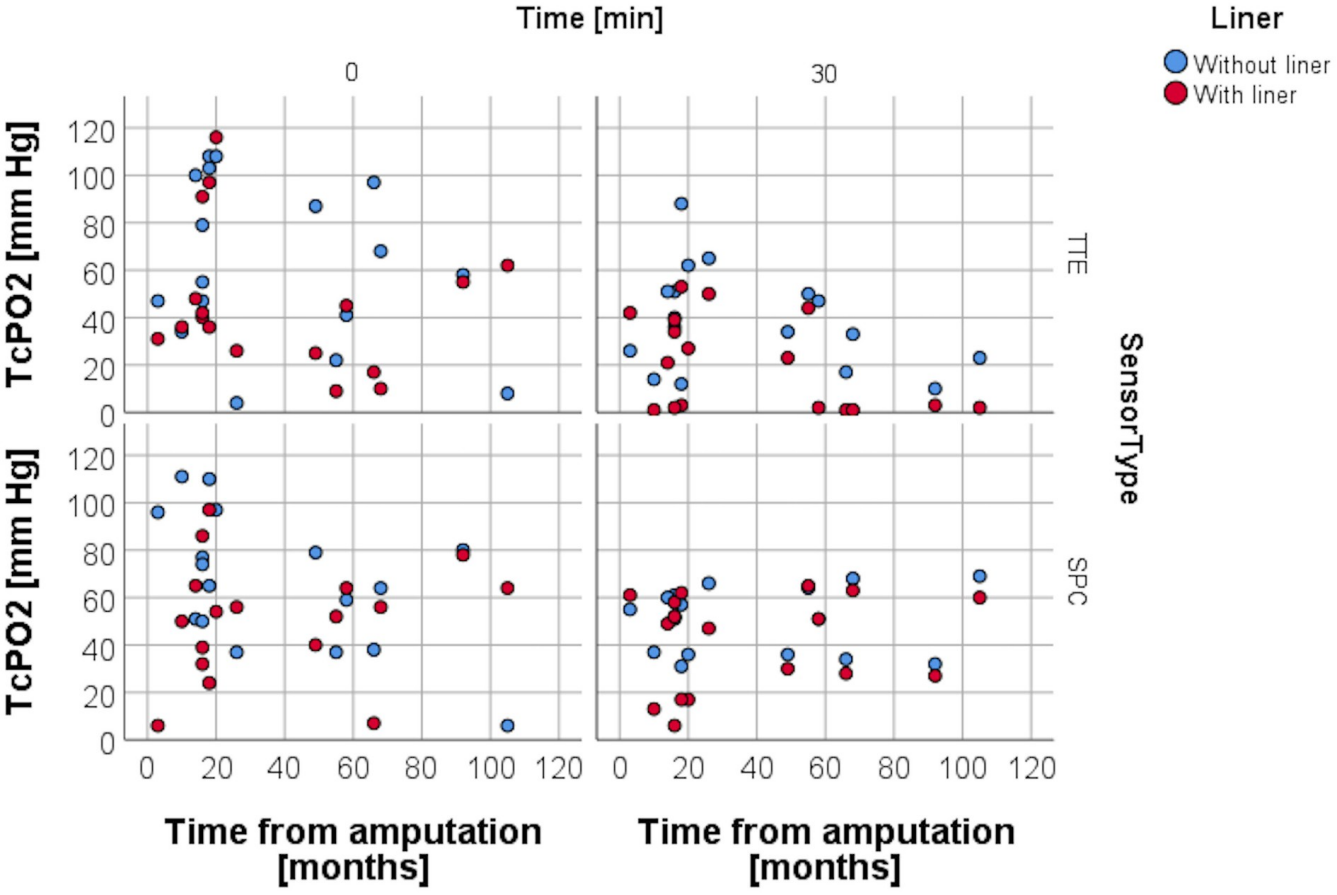
TcPO2 levels and time from amputation. Scattergram depicting distribution of TcPO2 levels with and without liner wear depending on the time from amputation.

RLP was assessed by using a VAS to evaluate the average amount of pain within the last four weeks. Spearman rank correlation analysis carried out for resting TcPO2 and the VAS obtained for RLP (Fig 6) at study entry was non-significant for both sensors in both conditions, as was the analysis for resting TcPO2 and age (Fig 7), daily prosthesis use (Fig 8), and for time from amputation (Fig 9).

### Resting TcPO2 and sex

**Fig 10:**
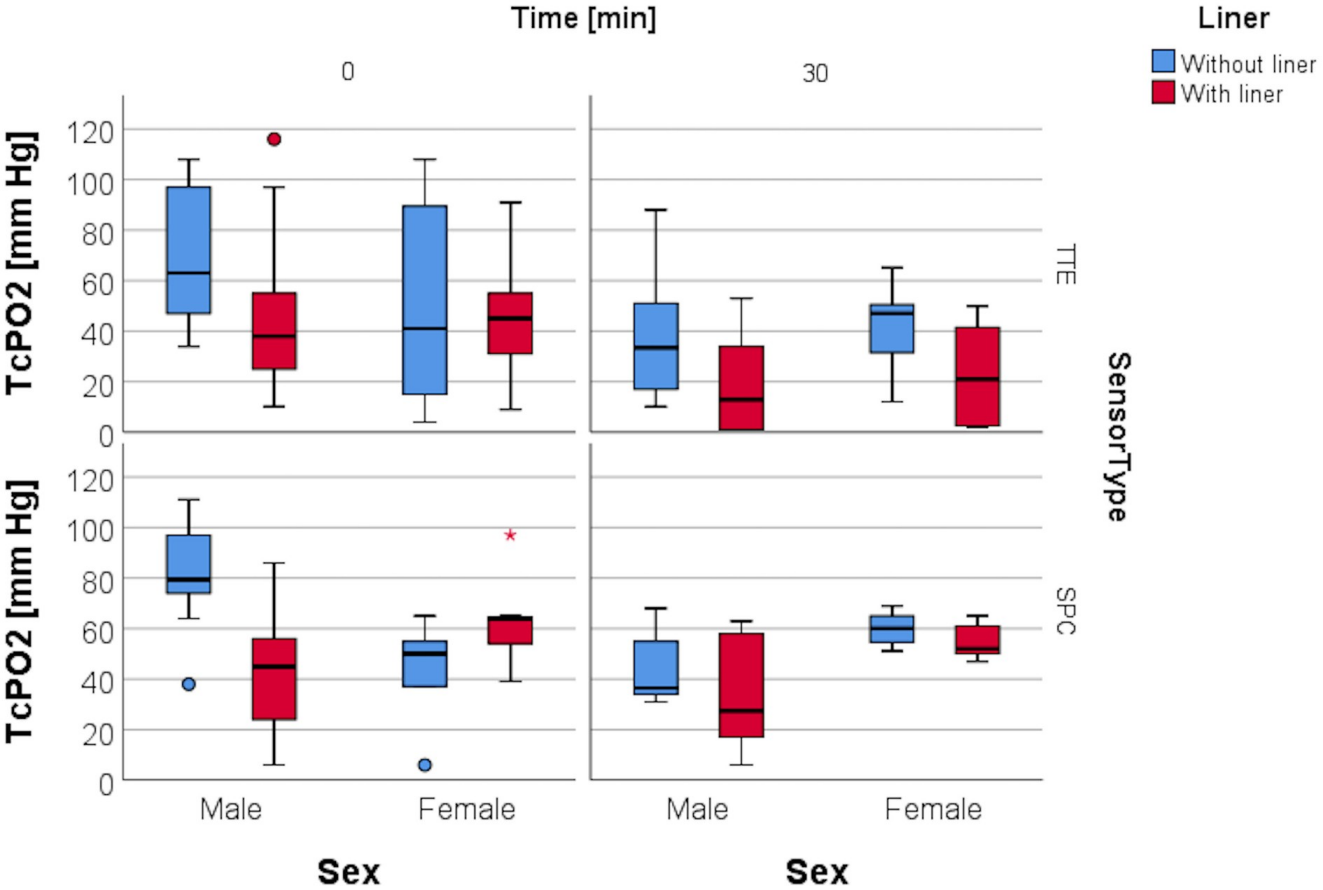
TcPO2 levels and sex. Boxplot depicting median TcPO2 levels of both sensors for T0 and T30 depending on sex with and without liner.

Mann-Whitney U testing was used to evaluate the association of resting TcPO2-levels and sex. For Sensor TTE, there were no statistically significant differences in TcPO2 between women and men whether a liner was worn or not. For Sensor SPC however, the results varied: without a liner, women had a significantly higher TcPO2 level at baseline (difference in medians = 29.5 mmHg, p = 0.003). This difference remained at T30: the median value for men was 36.5 mmHg, whereas the median value for women was 60.0 mmHg(p = 0.028). But when wearing a liner, no differences between men and women were observed at T0 (difference in medians = 19.0 mmHg, p = 0.107), or at T30 (difference in medians = 24.5 mmHg, p = 0.051) (Fig 10).

### Resting TcPO2 and variables K-levels and revision surgery

A one-way ANOVA was conducted to assess the effects of K-level and revision surgery on resting TcPO2 levels. Analyses showed no statistically significant difference for TcPO2 at baseline over the different types of K-level (with liner: TTE: p = 0.117, SPC: p = 0.453, no liner: TTE: p = 0.208, SPC: p = 0.906). Thus activity level did not have an influence on resting TcPO2. Also, presence of revision surgery at baseline did not have a statistically significant effect on resting TcPO2-levels (with liner: TTE: p = 0.257, SPC: p = 0.950, no liner: TTE: p = 0.528, SPC: p = 0.801).

## Discussion

Despite an interdisciplinary approach for each patient, our clinic sees a substantial number of persons with a TTA amputation who experience RLP for longer than three hours daily. No specific somatic pathology, such as osteitis, osteomyelitis, abscess, or poor bone shaping is at the cause of this pain. Further, common issues related to the residual limb skin that can cause RLP such as soft tissue surplus, skin lesions, ulcers, neuroma, heamatoma or skin allergies are also not the cause. Poor prosthetic fitting or adjacent joint degeneration also do not account for these cases. However, many of these patients have PAD. Two case reports link PAD with RLP [8, 9], and both highlight the need for an increased focus on possible residual limb claudication. We were therefore interested in analyzing if reduced local microperfusion induced by the compression exercised by a liner on itself was related to RLP. Such a decreased microperfusion would show as a decreased resting TcPO2 level.

Our results revealed that resting TcPO2 levels indeed decreased significantly when participants used their liner, indicating reduced perfusion. A significant drop in resting TcPO2 levels was observed within the first 10 minutes of liner usage, i.e. from T0, the baseline measure, to T10. After this drop, resting TcPO2 levels stabilized, and no further significant decreases could be detected. These results confirm our hypothesis that compression induced by a liner alone reduces microperfusion immediately after liner application. Microperfusion might be able to adapt to the increased compression, as indicated by the slight increase in TcPO2 levels between T10 and T20. Thereafter, the reduction remains relatively constant over the 30-minute frame, in measurements taken at T20 and T30.

However, a similar pattern in resting TcPO2 levels decrease was also observed when participants did not use their liner. This finding implicates that, independent of liner usage, a decrease in resting TcPO2 level occurs in supine position over the 30 minutes in which recordings were taken, albeit the decreases with a donned liner were larger.

Results similar to ours were documented by Rink et al. [26] Interested in residual skin health of persons with a TTA, they assessed resting TcPO2 levels, soft tissue perfusion through laser Doppler flowmetry, and skin temperature. All three measurements were taken at the posterior side of the residual limb, with TcPO2 measured most distally. The mean resting TcPO2 for their 5 participants was 57.8 mmHg ± 9.2, a value averaged from a 1-minute continuous recording taken after an accommodation period of 15 minutes. Because their TcPO2 sensor location was posterior and values were averaged from a continuous recording, a direct comparison with our results is not possible. Closest to their location would be our SPC sensor: resting TcPO2 for the donned liner at T0, our baseline measure taken directly after an accommodation period of 15 minutes, was 51.6 mmHg ± 22.7. This result is point-based, established by 20 participants, with a much larger standard deviation. Our next measurement occurred ten minutes later at T10, showing a much-reduced value of 40 mmHg ± 14.5. The method used by Rink et al. to measure resting TcPO2 levels also differed from ours. To minimize probe and sensor interference, Rink et al. created a silicone probe holder, in which the probe, sensors, and corresponding electrical cables were embedded evenly to create a flush surface against the residual skin [26]. Since the outer side of the probe holder was also smooth, it created a slightly bulkier calf, which could be compensated for by a larger liner. With their snug set-up, their approach most likely comes closest to a “real” situation in which a liner is donned.

The TcPO2 recordings of Sensor SPC during liner wear were higher than recordings in the same condition at Sensor TTE. Measurement location influences TcPO2 levels considerably. The angiosome concept first introduced by Taylor and Palmer [36] provides a possible explanation: while Sensor TTE was in the territory of the posterior tibial artery, Sensor SPC was placed in the junction of the sural and the descendent genicular arteries. This dual blood supply may have led to the higher TcPO2 values observed with Sensor SPC. Further, the remaining peroneal muscle bellies might be thicker than the remaining gastrocnemius muscle tissue underlying the tibial tip, especially in patients with PAD, in whom many surgeons remove the soleus muscle.

Because of the limited literature available for comparison of results, we turned to results obtained by thermography, as they correlate well with resting TcPO2 measurements [37]. Thermographic results from the literature also reveal high dependency on location, similar to our results. Peery et al. showed an overall increase in skin temperature after a resting period of 15 minutes with a donned prosthesis in their participants with a TTA [38]. Comparison of their sensor location and our sensor placement indicates they registered a decrease in temperature at similar locations to ours [38, 39]. The overall increase in temperature observed by Peery et al. does not seem to correlate with blood flow, but instead could be a thermal response to pressure with shear [40], as their participants donned their prosthesis while resting measurements were undertaken. Shear loads seem to further decrease TcPO2 levels when applied additionally to normal loads [41].

Unfortunately, we could not demonstrate a link between resting TcPO2 levels and RLP. One of the inclusion criteria for our participants was having RLP for more than three hours per day when wearing the prosthesis. Thus, RLP affects our study population considerably. RLP elicited due to an ill-fitted prosthetic socket can be excluded, as all sockets were well fitted and did not create any challenges for any of the participants. But RLP as a result of prosthetic alignment cannot be ruled out. Kobayashi et al. demonstrated a significant relationship between even small malalignments of 2° in abduction and adduction and the socket reaction moment impulse [42]. As they did not measure in-socket pressures, it is unknown if the recorded socket reaction moment led to an increase in socket pressure. These pressures could be small and absorbed by the liners in such a way that no pressure marks would be visible on the residual limb skin. However, increased pressure not shown as a pressure mark on the skin could nevertheless have an influence on tissue perfusion.

Not finding an association between reduced resting TcPO2 and pain is not an indicator that no relationship exists. The relationship between symptom intensity and ischemia in PDA is complex and often highly individual. For example, TcPO2 measures recorded during treadmill walking have shown that severe ischemia and pain do not need to occur simultaneously, and that pain can disappear despite persistent significant ischemia, independent of location [43, 44]. Nevertheless, Schorr et al. reported that roughly 69% of 120 treadmill exercise tests needed to be stopped due to pain [44]. Literature on how active TcPO2 levels affect pain in persons with a TTA while walking is lacking.

The extent of daily liner usage, mobility level (K-Level), age, revision surgery, and smoking status did not have a significant impact on TcPO2 levels. Women had significantly higher TcPO2 levels after 30 minutes of liner usage for sensor SPC than men, meaning that their soft tissue perfusion as measured by TcPO2 was better at this location than that of the men. As we are not aware of sex differences in the angiosome concept, this result could either be due to coincidence or an indication of a different amount of soft tissue shrinkage in women. To date, the literature reports no differences in amount of soft tissue shrinkage between men and women [45]. Alternatively, this result may indeed be a sign of angiosome sex differences.

### Study limitations

This study has several limitations. All tests were made during 1 and 5 p.m. due to logistical restrictions. While participants were informed to discontinue their liner and prosthesis usage on the test day until after testing was conducted, lack of compliance might have influenced TcPO2 starting levels.

All measurements were made during rest and with the participants in supine position. Because no control measurements were taken with participants, for example in sitting position with the residual limb in a vertical rather than a horizontal position, we cannot determine how much of an influence the supine position had on the TcPO2 levels. The effect of both standing and walking on TcPO2 levels while using a liner remains unclear. For persons without an amputation, exercise TcPO2 levels differ from resting TcPO2 levels by up to 50 mmHg [46–49].

Our method to punch sensor-sized holes into the liner to reduce liner-induced pressure onto the sensors may not represent the situation of a non-modified liner. How much these holes affect the readings is difficult to assess. Lenz and Bush described the effects of normal and shear forces at the socket/liner interface of a donned transtibial prosthesis in a case study [50]. Utmost care was taken to create an exact duplicate of the participant’s current prosthesis. In their last test condition (condition 3) a large hole (> 4 cm in diameter) was cut into the gel liner to allow for load cell measurements taken directly at the skin level. Comparing condition 3 to condition 1, where force measurements were taken at the prosthetic wall, their results revealed a significant decrease in normal and shear forces throughout the gait cycle with condition 3. Applying their results to our situation, holes within the liner would reduce forces exerted by the liner onto the residual limb, thus also reducing compression. However, at both sensors and at all time points except baseline, a significant TcPO2 reduction was observed when wearing the liner. This indicates that the liner’s effect was still present, despite its small holes. Nevertheless, an underreporting of the actual values measured in our liner condition is possible.

Caution needs also to be taken when generalizing our results to other types of liner, as different liner materials and their effects on in-vivo performance are not well understood [51].

Additionally, liner sizes are not continuous. Rather, different residual limb circumferences are bundled into size categories. Within a given category, pressure may vary depending on whether the residual limb circumference is at the border of the next category or is well within the category. Thus, liner size is a potential confounding variable that may have contributed to the larger variability observed in the condition with the liner donned.

Finally, pain was not measured at timepoints T0, T10, T20 and T30, but at study entry. Potential changes in RLP should be evaluated in further studies to determine possible pain changes during measurements.

## Conclusion

Compared to resting TcPO2 with no silicone liner, a significantly reduced resting TcPO2 was observed with the liner donned within our specific population of mainly persons affected with PAD. However, no association could be found between resting TcPO2 and pain. Of all the variables tested for a potential influence on resting TcPO2 levels (i.e., smoking, age, sex, mobility level, duration of daily liner usage, and surgical revision), only sex was significant: women had a significantly higher resting TcPO2 level for Sensor SPC at T0 and T30. Further studies are needed to investigate if the same applies when TcPO2 levels are measured during dynamic situations.

## Supporting information

S1 File (TXT)
